# Autism and attachment disorder symptoms in the general population: Prevalence, overlap, and burden

**DOI:** 10.1177/2516103220902778

**Published:** 2020-02-17

**Authors:** Helen Minnis, Claudia-Martina Messow, Alex McConnachie, Paul Bradshaw, Andrew Briggs, Philip Wilson, Christopher Gillberg

**Affiliations:** 1 University of Glasgow, UK; 2 ScotCen Social Research, UK; 3 University of Aberdeen, UK

**Keywords:** Autism spectrum disorder, disinhibited social engagement disorder, general population, reactive attachment disorder

## Abstract

**Background:**

Co-occurring trauma-related and neurodevelopmental problems are common in maltreated children. In population research and clinical practice, these tend to be considered separately. Overlapping health problems, that is, “multi-morbidity,” in adulthood is associated with increased service burden and costs, but this has not been investigated in childhood.

**Methods:**

Using well-validated parent-report questionnaires, we examined the overlap between symptoms of the neurodevelopmental disorder autism (autism spectrum disorder, ASD) and symptoms of the trauma- and stressor-related disorders (reactive attachment disorder [RAD] and disinhibited social engagement disorder [DSED]) in a representative general population sample of over 3,300 children aged 5–6 years of age. We investigated sociodemographic factors, service burden, and costs in association with these problems when considered separately and when co-occurring.

**Results:**

Nearly 2% of this population had symptoms suggestive of both ASD and RAD/DSED. High symptom scores for ASD were associated with male gender, (younger) age of mother at birth, and being in a single-parent family, while high symptom scores for RAD/DSED were associated with (younger) age of mother at birth, being in a single-parent family, and the number of accidents reported. Service use costs per likely case of both ASD and RAD/DSED in the preschool years were increased by £348.62 (95% confidence interval 121.04–391.11)—nearly double the costs of ASD alone.

**Conclusions:**

There is considerable overlap between symptoms of ASD and RAD/DSED in the general population, indicating that multi-morbidity is already present in childhood and is associated with increased service use and costs even in the preschool years.

## Background

Children who have been abused and neglected are much more likely than their peers to have symptoms of neurodevelopmental problems such as autism spectrum disorder(ASD), attention deficit hyperactivity disorder (ADHD), and intellectual disabilities (Dinkler et al., 2017). In turn, children with neurodevelopmental disorders such as ASD, ADHD, and intellectual disability are at higher risk than their peers of experiencing maltreatment (McDonnell et al., 2019; Stern et al., 2018). The symptoms of these neurodevelopmental problems can increase parental stress (Miranda et al., 2015) and place children at risk of being maltreated.

We, and others, have investigated the causal links between neurodevelopmental problems and maltreatment. Behavioral genetic findings suggest that maltreatment does not cause neurodevelopmental problems in abused and neglected children (Dinkler et al., 2017) and longitudinal research has now shown that neurodevelopmental problems, including ADHD and intellectual disability, occur *before* maltreatment in the life cycle(Danese et al., 2016; Stern et al., 2018). Together, these findings suggest that neurodevelopmental problems and maltreatment need to be considered together—and that neurodevelopmental problems might be important risk factors for child abuse and neglect.

However, children who have experienced maltreatment are also at risk of developing problems that directly arise from abuse and neglect. Two “trauma & stressor related disorders” are described in Diagnostic and Statistical Manual (DSM)-5: disinhibited social engagement disorder (DSED), characterized by indiscriminate behaviors and reactive attachment disorder (RAD), characterized by failure to seek/accept comfort and emotionally withdrawn behaviors (American Psychiatric Association, 2013). At the time the data for this study was collected, RAD and DSED were considered as different manifestations of the same disorder, whereas they are now understood as different disorders, probably with differing etiology, though often co-occurring (Zeanah & Gleason, 2015). Throughout the article, we use the term “RAD/DSED” when discussing studies in which the two disorders were examined together. RAD and DSED are serious disorders of social functioning (Gleason et al., 2011) that are thought to have a poor long-term prognosis if untreated (Rutter et al., 2009). Both psychiatric classification systems (International Classification of Diseases (ICD)-10 and (DSM-V)) state that the diagnosis should *only* be made if there is a history of serious early childhood maltreatment (American Psychiatric Association, 2013; World Health Organization [WHO], 1993).

Traditionally, these “trauma & stressor related problems” (assumed caused by maltreatment) (Zeanah et al., 2016) and neurodevelopmental problems (which are highly heritable; Taylor et al., 2015) have been regarded as entirely separate entities. Both the DSM and ICD classification systems recommend that, when assessing for RAD and DSED, neurodevelopmental disorders such as ASD should be diagnoses of exclusion (American Psychiatric Association, 2013; WHO, 2010, 2016). Yet such exclusionary diagnostic systems are being increasingly challenged in child psychiatry since overlap in psychiatric syndromes is the rule (Gillberg, 2010; Minnis, 2013) and a full understanding of a child’s profile is essential for appropriate management and treatment planning (Hunt & Rodwell, 2018). In adult health, there is an increasing focus on the co-occurrence of health problems: “multi-morbidity,” the presence of two or more disorders, has been shown to place a particular burden on health and social care services (Barnett et al., 2012) and to be associated in adulthood with adverse childhood experiences such as abuse and neglect (Anda et al., 2006). There has been little research on multi-morbidity in early childhood (Chau et al., 2013) especially in the context of abuse and neglect where it is most likely to occur (Dinkler et al., 2017) and when the effects might be most modifiable (Kim & Cicchetti, 2010). We know nothing about any associated burden on individuals, their families, or on health and other services. ASD is associated with high societal costs related to intense use of public services including education (Barrett et al., 2012; Knapp et al., 2009) but only one study has previously examined service use and costs in relation to RAD and DSED symptoms (Minnis et al., 2006) and none have examined both neurodevelopmental and trauma- and stressor-related disorders together.

Theoretically, there is no reason why neurodevelopmental problems such as ASD and trauma- and stressor-related problems such as RAD and DSED could not occur together: A child with heritable ASD could experience maltreatment in the same way as a child without and, in addition, children with inherent deficits in social communication or other symptoms of neurodevelopmental problems may be more at risk of being maltreated if in a vulnerable family (Gul & Gurkan, 2018; Sullivan & Knutson, 2000).We have already shown, in clinic-based research, that ASD and RAD/DSED can occur together in maltreated children (Kocovska et al., 2012), but the prevalence of co-occurring ASD and RAD/DSED in the population is unknown. Co-occurrence of RAD/DSED and ASD might place children at particularly high risk of current and future physical and mental illness, as has been found when multi-morbidity occurs in adults (Barnett et al., 2012).

The population prevalence of ASD is around 1.1% (Baird et al., 2006), although estimates vary between geographical locations (Parner et al., 2011). We established the prevalence of RAD/DSED (Minnis et al., 2013) and it was similar to the prevalence of ASDs at 1.4% (Minnis et al., 2013). In this study, we have the first opportunity to investigate whether overlap between ASD and RAD/DSED is present in the general population at early school age and whether overlapping ASD and RAD/DSED is associated with an increased burden to families and services.

Our research questions were the following:

- What is the prevalence of ASD and RAD/DSED symptoms among 6-year-old Scottish children?
- What is the overlap between ASD and RAD/DSED symptoms among 6-year-old Scottish children?
- What are the sociodemographic features associated with the two conditions?
- What are the health-care burdens and costs associated with the two conditions?

## Methods

### Ethics

The initial sweep of data collection was subject to medical ethical review by the West of Scotland “A” MREC committee (application reference: 04/M RE 1 0/59). Subsequent sweeps have been reviewed via substantial amendments submitted to the same committee. The study has therefore been performed in accordance with the ethical standards laid down in the 1964 Declaration of Helsinki and its later amendments. At the outset of the study, participants were provided with study details before written, informed consent was collected. Participants are regularly provided with updated study information and consent for re-contact and ongoing participation is collected on a continuous basis at all sweeps of data collection.

### Sample

The growing up in Scotland (GUS) is a national sample including a birth cohort (*n* = 4,191; 80.3% of original cohort), born in 2004/2005. It includes face-to-face interviews with parents annually until age 5, then at stages of interest. In order to investigate the patterning and correlates of ASD and *ASD and* RAD/DSED symptoms in the Scottish population, screening questionnaires for these disorders were included in the sixth sweep of GUS (2010–2011), when the children were approximately 6 years old.

The methodology for collecting data on questionnaires for ASD and RAD/DSED, the Autism Spectrum Screening Questionnaire (ASSQ) and Relationship Problems Questionnaire (RPQ) (see below for details), was piloted with 96 children in GUS. The parental response rate in the pilot was 74% and there were no major concerns regarding the questionnaires. Very minor modifications (not affecting the meaning of core concepts) were made to the ASSQ for this Scottish population.

In the main-stage survey (data collected as part of the 2010/2011 sweep of GUS), parents were sent copies of the ASSQ and RPQ (see below) with the “advance letter” that was sent out approximately 1 week prior to the home interview and were asked to complete them in advance. If the questionnaire had been completed, it was collected by the interviewer on the day of interview, or at a later home visit or by post if not completed on the day. Completed questionnaires were transferred to the Robertson Centre for Biostatistics for data processing and analysis.

The GUS population was derived from Child Benefit records (at that time a universal benefit taken up by around 97% of eligible families). Stratified cluster sampling was used to derive a nationally representative sample. Child Benefit records indicate that among the eligible population, 6% of children were born to mothers aged under 20 and 18% lived in an area in the most deprived quintile of the Scottish Index of Multiple Deprivation (SIMD; see below). After applying weights to adjust for nonresponse and sample selection, 8% of the GUS sample at Sweep 6 had mothers aged under 20 at birth and 19% lived in an area in the most deprived quintile. As such, the GUS sample is considered to be representative of the study population from which it is drawn.

### Measures

The ASSQ is a 27-item screening instrument for symptoms of ASD with versions for parents and teachers and three possible responses “no,” “somewhat,” and “yes.” In this study, the parent version was used. Because it is completed by lay informants and items can be interpreted subjectively, it is not intended as a diagnostic measure, but simply as a measure that indicates which children require further assessment for ASD. It has been widely used and validated in general population research, including in the Barn i Bergen study—a longitudinal cohort study of 10,000 school-age Norwegian children (Posserud et al., 2006) and in a diagnostic study focusing on Asperger’s syndrome involving over 4,400 Finnish school children (Mattila et al., 2007). It has good test–retest and inter-rater reliability and the optimum cutoff point (giving the best balance of sensitivity versus specificity) has been determined at a score of 19 on the parent-rated ASSQ (Ehlers et al., 1999). ASD high scorer status was therefore defined in this study as those children with a score on the ASSQ of 19 or above (Ehlers et al., 1999).

The RPQ is a 10-item questionnaire, with versions for parents and teachers, for RAD/DSED symptoms (Minnis et al., 2007). It has four possible responses (“Not at all like my child,” “A bit like my child,” “Like my child”, and “Exactly like my child”) scored 0, 1, 2, and 3. In this study, the parent version was used. The RPQ was completed by over 13,000 parents in a large general population twin sample, the Twins Early Development (TEDS) study (Minnis et al., 2007). It was found to have good internal consistency (Cronbach’s α 0.85). This work demonstrated that RAD/DSED symptoms are present in the general population and are associated with harsh parenting. The RPQ has also been used in a population-based diagnostic study involving over 1,600 children and the optimum cutoff point (giving the best balance of sensitivity versus specificity) has been determined at a score of 7 on either the parent or teacher-rated RPQ. RAD/DSED high scorer status was therefore defined in this study as those children with a score on the RPQ of 7 or above (Minnis et al., 2013).

In order to ensure that any symptomatic overlap between the ASSQ and RPQ was not simply due to overlap between the questionnaires, we performed factor analysis.

Deprivation in GUS was measured by SIMD. SIMD ranks small postcode areas in quintiles according to deprivation from 1 = *Most deprived* to 5 = *Least deprived*. The SIMD is derived from 38 indicators across 7 domains: income, employment, health, education, skills and training, housing, and geographic access and crime (www2.Scot.gov.uk/Topics/Statistics/SIMD/BackgroundMethodology).

Cost for service use was derived from the reported number of contacts with health-care professionals over the 6 months preceding data collection. This information was collected at Sweeps 2–4, when the child was approximately 2, 3, and 4 years old. Costs for each health-care contact were taken from Unit Costs of Health and Social Care (PSSRU, 2018).

Additional sociodemographic and predictor variables were also derived from questions asked in the various main GUS face-to-face interviews conducted when the child was aged between 10 months and 6 years. For example, caregivers were routinely asked how many accidents or injuries requiring medical attention the child had in the last 12 months. Where any such accidents were reported, parents were then asked if the child attended accident and emergency as a result. Parents were also asked if they had attended accident and emergency in the previous 6 months to seek treatment for or advice on other issues with the child’s health. To measure the use of smacking, caregivers were asked to choose which disciplinary techniques they had ever used by referring to options on a card. For each option, a response of *mentioned* or *not mentioned* was recorded.

### Statistical analysis

Confidence intervals (CIs) for prevalence rates have been calculated using the normal approximation. Sociodemographic variables have been summarized as number of children per category and percentage of these scoring positive, and their relation to caseness has been assessed through Fisher’s exact tests. Values of potentially associated variables at Sweep 6 (closest to the time of the questionnaire) have been used, except for parenting variables (smacking and shouting) and number of accidents, where all available time points have been used.

For ASD, RAD/DSED, and ASD + RAD/DSED, a multivariate logistic regression model predicting caseness was derived by forward selection from all predictors identified in the univariate analyses. In addition, analyses were repeated using weightings to account for study attrition. Details about the weights can be found in the GUS study data documentation (Bradshaw et al., 2011). Since differences between the weighted and the unweighted analyses were small, we only present the unweighted results.

For predicting costs, values of the predictor variables at the same sweep as the cost data have been used, except for education where values at Sweep 6 were used, assuming that the important education factor was the maximal level of education achieved rather than education at the time of the sweep.

The shape of the relation between questionnaire scores and cost was assessed predicting cost from thin plate regression splines of the questionnaire scores (these aim to give a smooth fit of the data) (Wood, 2003).

Cost was modeled using generalized mixed models assuming a log link and gamma variance. Models were constructed including ASSQ score or RPQ score or both scores, or both scores and their interaction. Similar models were constructed using likely caseness rather than questionnaire scores. Other variables were selected in forward stepwise variable selection from age of child at each sweep, child sex, age of mother at birth, household level of education at Sweep 6, family type, combined household employment and family type, number of adults in household, number of children in household, income, urban-rural classification, and SIMD.

Estimates for costs for health-care contacts over the period from the age of 18 months to 4 years have been derived from the above model for caseness using the method of recycled prediction (Glick et al., 2007). We predicted cost differences between:

— children with likely ASD and other children (including children with likely RAD/DSED),
— children with likely RAD/DSED and other children (including children with likely ASD),
— children with likely ASD or likely RAD/DSED or both and children without ASD and RAD/DSED, and
— children with both likely ASD and likely RAD/DSED and children without ASD and RAD/DSED.

All results of regression analyses shown here have been carried out on unweighted data, assuming that the relationship of one variable with another should not be affected by attrition. Analyses were repeated using weights and differences were found to be small (available on request).

All analyses have been carried out in R version 3.0.1 (R Core Team. (2013). *R: A language and environment for statistical computing*. R Foundation for Statistical Computing, Vienna, Austria. http://www.R-project.org/). Since this is an exploratory analysis, *p* values have not been corrected for multiple testing and have to be considered as descriptive.

## Results

Of the target sample of 4,191, 3,355 (80%) questionnaires were returned. For incomplete questionnaires, scores were prorated if at least 60% of the items had been completed. This resulted in 3,349 (79.9% of the target sample) questionnaires that could be analyzed. During data cleaning, 18 questionnaires (0.5% of the sufficiently completed ones) were removed because they had maximum scores in either ASSQ or RPQ and a score of 0 in the other, which was assumed to be highly unlikely. The remaining 3,331 questionnaires (79.5% of the target sample) were used for the analysis.

Of this final sample, 49.0% are girls and the mean age is 70.2 months (5.85 years), the age range is 69–72 months (5.75–6.00 years). Our factor analysis (results available on request) showed virtually no overlap between the ASSQ and RPQ. We have presented our results according to the research questions:

- What is the prevalence of ASD and RAD/DSED symptoms among 6-year-old Scottish children?

Of the 3,331 children with complete data, 139 (4.2%, 95% CI 3.5–4.9%) screened positive for ASD (i.e., had an ASSQ score of 19 or more). Using the metrics from the Finnish sample as a guide, in which 26% of screen-positive children had an actual ASD diagnosis (Mattila et al., 2007), this might translate to approximately 36.1 cases of ASD in this population, or a prevalence of approximately 1%.

Of the 3,331 children with complete data, 154 (4.6%, 95% CI 3.9–5.3%) screened positive for RAD/DSED. Using the metrics from the Minnis et al sample as a guide (Minnis et al., 2013), in which 16.04% of screen-positive children had an actual RAD/DSED diagnosis using gold-standard diagnostic instruments, this might translate to approximately 24.7 cases of RAD/DSED in this population, or a prevalence of approximately 0.7%.

- What is the overlap between ASD and RAD/DSED among 6-year-old Scottish children?

**Figure 1.**
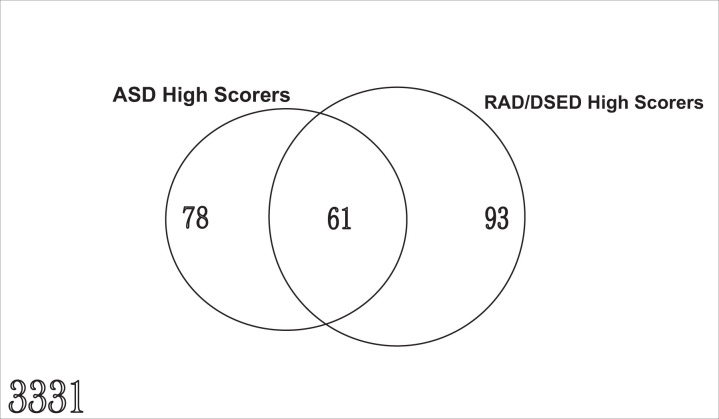
Number of children in each high scoring group and overlap.

Of the 3,331 children with data available for both questionnaires, 61 (1.8%) were high scorers for both ASD and RAD/DSED. Of the RAD/DSED high scorers, 39.6% were also ASD high scorers and of the ASD high scorers, 43.9% were also RAD/DSED high scorers (see Figure 1 and Supplemental Table 1, Web Appendix).

- What are the sociodemographic features associated with the two conditions?

*ASD high scorers* (see Supplemental Tables 1 and 2, Web Appendix): In univariate analyses, no relevant associations were observed between prevalence of ASD high scoring and parental educational level, household income, or level of deprivation. ASD symptoms were not associated with parental endorsement of smacking as a form of discipline.

The univariate analyses did show that ASD high scoring was more common in boys, lone-parent families, families with younger mothers, and families with more than three children. High ASD scorers were also reported to have poorer general health, and to have more accidents (see Supplemental Tables 1 and 2, Web Appendix).

In multivariate analyses (see Table 1), ASD high scoring was associated with male gender, (younger) age of mother at birth, and being in a single-parent family but number of accidents was no longer a predictor.

**Table 1.** Multivariate predictors of ASD, RAD/DSED, and overlap.

Predictor		ASD	RAD/DSED	ASD + RAD/DSED
		OR (95% CI), *p* value	OR (95% CI), *p* value	OR (95% CI), *p* value
Sex of child	Male	Reference		Reference
	Female	0.58 (0.41, 0.83), *p* = 0.003		0.57 (0.33, 0.98), *p* = 0.041
Age of mother at birth	<20	Reference	Reference	Reference
	20–29	0.68 (0.37, 1.25), *p* = 0.216	0.63 (0.34, 1.14), *p* = 0.126	0.73 (0.28, 1.92), *p* = 0.523
	30–39	0.32 (0.17, 0.61), *p* < 0.001	0.37 (0.20, 0.69), *p* = 0.002	0.37 (0.14, 1.00), *p* = 0.049
	≥40	0.20 (0.04, 0.89), *p* = 0.035	0.58 (0.21, 1.57), *p* = 0.286	0.25 (0.03, 2.16), *p* = 0.207
Number of accidents reported (Sweeps 1–6)	None		Reference	
	1–2		0.88 (0.61, 1.28), *p* = 0.510	
	3–5		1.36 (0.83, 2.23), *p* = 0.227	
	6 or more		4.75 (2.15, 10.49), *p* < 0.001	
Family type (Sweep 6)	Lone parent	Reference	Reference	
	Couple family	0.60 (0.40, 0.90), *p* = 0.014	0.62 (0.42, 0.92), *p* = 0.018	

*Note*. Associations reported as OR with 95% CI and *p* value. Models identified by backward selection from all predictors are presented in Supplemental Tables 1 and 2, Web Appendix. OR: odds ratio; CI: confidence interval; ASD: autism spectrum disorder; RAD: reactive attachment disorder; DSED: disinhibited social engagement disorder.

*RAD/DSED high scorers* (see Supplemental Tables 1 and 2, Web Appendix): In univariate analyses, no relevant associations were observed between prevalence of RAD/DSED high scoring and parental educational level, household income, or level of deprivation. RAD/DSED symptoms were not associated with parental endorsement of smacking as a form of discipline.

The univariate analyses did show that prevalence of RAD/DSED high scoring was associated with male gender, lone parenthood, more than three children, and younger mother’s age at birth (see Supplemental Table 1, Web Appendix). There was also an association with poorer general health in the child and more accidents (see Supplemental Table 2, Web Appendix).

In multivariate analyses (see Table 1), RAD/DSED high scoring was not associated with gender, but was associated with (younger) age of mother at birth, number of accidents, and being in a single-parent family.

*High scorers for both ASD and RAD/DSED* (see Supplemental Tables 1 and 2, Web Appendix): In univariate analyses, no relevant associations were observed between prevalence of high scoring for both disorders and parental educational level, household income, level of deprivation, number of adults or children in the household, or lone-parent status. Symptoms of both disorders were not associated with parental endorsement of smacking as a form of discipline.

The univariate analyses did show that the prevalence of high scoring for both disorders was associated with male gender and younger mother’s age at birth. There was also an association with poorer general health and more accidents (see Supplemental Tables 1 and 2, Web Appendix).

In multivariate analyses (see Table 1), high scoring for both disorders was associated with gender and (younger) age of mother at birth.

- What are the healthcare burdens and costs associated with the two conditions?

**Figure 2.**
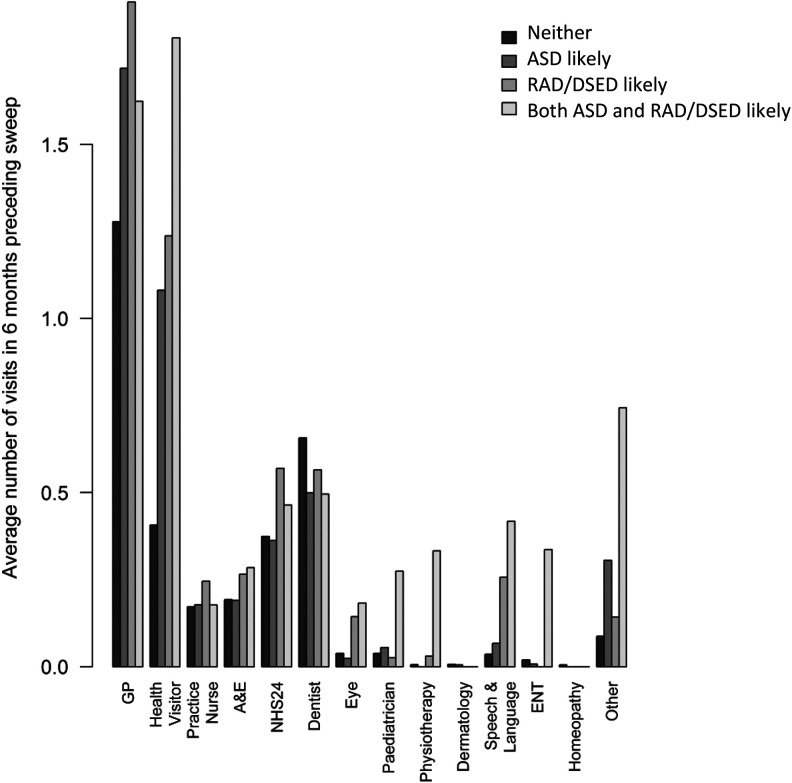
Health service use.

Figure 2 shows the average number of health-care professional visits in the 6 months preceding the sweep, by likely caseness and by type of health-care professional, and the distribution of the costs associated with health-care professional contacts, by sweep and likely caseness. Being a high scorer for both ASD and RAD/DSED symptoms was associated with more contacts in the preschool period with various health professionals including the health visitor, pediatrician, physiotherapist, and speech and language therapist. Children with high scores for either/both disorders were, however, less likely to visit the dentist.

Regression models (Supplemental Table 3, Web Appendix) predicted costs of being a high scorer (likely caseness) for ASD or RAD/DSED, that is, either including ASSQ high scorers only, including RPQ high scorers only or including both. Likely caseness was associated with higher costs. The number of accidents, child age, being male, and living in a more urban area increase costs. Using continuous questionnaire scores rather than likely caseness gives similar results.

The predicted increase in cost for health-care contacts over the period from 18 months to 4 years of age per ASD case was £178.87 (95% CI 47.85–408.14). The predicted increase in cost per RAD/DSED case was £228.76 (95% CI 87.14–443.83). The predicted increase in cost per case of ASD and RAD/DSED occurring together is £348.62 (95% CI 8.59–661.11).

## Discussion

Our findings have shown that neither ASD nor RAD/DSED symptoms are rare in the Scottish population and that overlap between these two clusters of symptoms is fairly common. These findings fit well with other recent findings in child and adolescent mental health: that symptomatic overlap between the various child psychiatric disorders is common (Gillberg, 2010) and the common genetic etiology that is now known to exist for a range of child neurodevelopmental disorders (Pettersson et al., 2013; Thapar et al., 2013).

Although this study focuses on symptoms and does not attempt to make diagnoses, our estimates of diagnostic prevalence may be useful in illuminating something about the nature of our population. Our estimate of likely ASD prevalence in the Scottish population of 6-year-olds (∼1%) is in accordance with other UK data sets (Baird et al., 2006). Our estimation of the likely prevalence of RAD/DSED in this population of Scottish 6-year-olds (∼ 0.7%) is approximately half of the prevalence of 1.4% estimated in a socioeconomically deprived Scottish population sample (Minnis et al., 2013). This is perhaps unsurprising as RAD/DSED is thought to only exist if there has been a history of abuse and neglect: although the overwhelming majority of people living in poverty do not abuse or neglect their children, rates of child maltreatment are higher in areas of high socioeconomic deprivation, possibly due to the stress associated with poverty (McSherry, 2004). However, this could perhaps be indicative of a methodological issue: although GUS is apparently representative of the general population, there may have been selective attrition of parents who were more likely to maltreat their children. This might suggest that the true prevalence of RAD/DSED in Scotland may be higher than our estimate suggests.

Some of the findings regarding ASD symptoms were unsurprising, for example, the male preponderance and the lack of association with sociodemographic variables and parenting, but there were some surprising results including that ASSQ scores were higher in children living in lone-parent families and who had mothers who were younger at their birth. Previous studies have not found associations with single parenthood (Montes & Halterman, 2007) and have found a higher prevalence of ASD in mothers who were *older* at the time of the baby’s birth: the seminal Kaiser Permanente study found a significant increase in ASD prevalence for every 10-year increase in maternal age, however, this study investigated the prevalence of ASD *diagnoses* (from Kaiser Permanente clinical outpatient records) (Croen et al., 2007). In the current study, we asked parents to report on *symptoms* of ASD and it is possible that younger mothers are less likely to seek services (and are hence less likely to get a diagnosis) than older mothers. Apparent effects of young age of the mother or lone parenthood may not be direct but could be the result of other factors such as maternal psychopathology, smoking, drugs use, obstetric problems, or poor medical assistance during pregnancy and it would be of interest to investigate these factors in future research.

Because RAD/DSED is thought to be caused by maltreatment, and maltreatment is known to be more prevalent in more deprived communities where social stressors are greater (Sidebotham et al., 2001), it is perhaps unsurprising that children with high scores on the RPQ were more likely to come from lone-parent families and to have mothers who were younger at the time of the child’s birth. It is, however, surprising that likely RAD/DSED caseness was not associated with employment, parental education, income or SIMD score. This is a reminder that child maltreatment—and RAD/DSED—can occur in all strata of society.

It is of note that, in this study, we did not find an association between RAD/DSED high scoring and parental endorsement of smacking but this may be a measurement problem in that parents may have been reluctant to admit to smacking their children in a climate where legal sanctions may apply for such behavior. In a previous large population (twin) study using the RPQ, there was a significant association between RAD/DSED symptoms and parental endorsement of using harsh parenting techniques—though the measure used for harsh parenting in that study was more detailed than that used in GUS (Minnis et al., 2007). The association between RAD/DSED high scoring and accidents leads us to speculate whether or not some of these injuries could have, in fact, been non-accidental, or that the higher frequency of accidents may have been related to the lack of supervision associated with neglect or because neglected children are less likely to ask for help.

The study is limited by the fact that we did not get data from 20.5% of the target population and may therefore have lost the most vulnerable families, hence underestimating the true prevalence of RAD/DSED symptoms as detailed above. This loss of the most vulnerable families may also have affected the social patterning of findings in ways it would be hard to predict. There may be other response biases within the data—for example, we cannot know whether younger or older mothers may complete questionnaires differently. Although the RPQ has been validated against diagnosis in a Scottish population (Minnis et al., 2013), the ASSQ has not so we would emphasize that our estimates for likely diagnosis would need to be further tested in a future study. Another limitation is the lack of diagnostic information: these data cannot tell us whether children with overlapping symptoms of ASD and RAD/DSED really do have both disorders or whether this is simply due to the symptoms, as reported by parents, being similar. Both DSM and ICD classification systems require a diagnosis of RAD and/or DSED only to be made if there has been maltreatment, but it is challenging to record child abuse in population research without jeopardizing response rates. Our previous research has shown that discrimination between ASD and RAD/DSED diagnosis is usually, but not always, possible in clinical practice(Davidson et al., 2015) and that co-occurrence of RAD/DSED and ASD is also possible (Kocovska et al., 2012). It will be important, in future research, to conduct full assessments of children who have high scores on both the ASSQ and RPQ within a population context to determine the prevalence of overlapping disorders.

Service use and costs of having both ASD and RAD/DSED in childhood are already increased by the time we investigated them in the preschool and early primary school years (additional cost per case of nearly £350). These costs seem modest compared to the enormous lifetime costs of ASD, recently estimated at approximately £1.23 million for someone with intellectual disability and approximately £0.80 million for someone with ASD without intellectual disability (Knapp et al., 2009). This suggests that costs multiply as the years progress and underlines the importance of intervening early to ameliorate this accruing burden on individuals and society.

Our findings suggest logical targets for early intervention. For example, interventions such as nurse–family partnership that target young parents and focus on the parent–infant relationship might be particularly well placed to prevent trauma- and stressor-related disorders emerging in infants/preschoolers with neurodevelopmental problems (Eckenrode et al., 2017). If proven successful, such early interventions might prevent increasing mental health burden accruing across the lifespan in these children.

## Conclusions

Almost 2% of preschool children in this Scottish general population cohort have symptoms of both ASD and RAD/DSED, suggesting they have both neurodevelopmental and trauma- and stressor-related problems. ASD nor RAD/DSED symptoms are associated with younger maternal age and being in a single-parent family and RAD/DSED is also associated with the number of reported accidents. Already in the preschool years, significantly increased service use and costs are associated with symptoms of both disorders, particularly when co-occurring.

## Supplemental material

Supplemental Material, Web_appendix_tables_2.12.19 - Autism and attachment disorder symptoms in the general population: Prevalence, overlap, and burden Supplemental Material, Web_appendix_tables_2.12.19 for Autism and attachment disorder symptoms in the general population: Prevalence, overlap, and burden by Helen Minnis, Claudia-Martina Messow, Alex McConnachie, Paul Bradshaw, Andrew Briggs, Philip Wilson and Christopher Gillberg in Developmental Child Welfare
