# Supplementary material for: Autism and attachment disorder symptoms in the general population: Prevalence, overlap, and burden
Source: Dev Child Welf. 2020 Feb 17;2(1):37–51. doi: 10.1177/2516103220902778 (PMC13021084; doi:10.1177/2516103220902778)
Supplement: Supplemental Material, Web_appendix_tables_2.12.19 - Autism and attachment disorder symptoms in the general population: Prevalence, overlap, and burden [file Web_appendix_tables_2.12.19.pdf]

Supplemental Table 1: Prevalence of ASD, RAD/DSED and overlap, in relation to household characteristics

		N	ASD		RAD/DSED		ASD + RAD/DSED	
			Prev (%)	p-value	Prev (%)	p-value	Prev (%)	p-value
All		3331	139 (4.2%)		154 (4.6%)		61 (1.8%)	
Number of adults in household (Sweep 6)	1	432	28 (6.5%)	p=0.032	30 (6.9%)	p=0.028	11 (2.5%)	p=0.306
	2	2643	99 (3.7%)		109 (4.1%)		44 (1.7%)	
	more than 2	254	12 (4.7%)		14 (5.5%)		6 (2.4%)	
Number of children in household (Sweep 6)	1	638	32 (5.0%)	p=0.015	35 (5.5%)	p=0.050	13 (2.0%)	p=0.183
	2	1809	60 (3.3%)		73 (4.0%)		31 (1.7%)	
	3	701	33 (4.7%)		30 (4.3%)		10 (1.4%)	
	more than 3	181	14 (7.7%)		15 (8.3%)		7 (3.9%)	
Family type (Sweep 6)	Lone parent	520	38 (7.3%)	p<0.001	39 (7.5%)	p=0.001	15 (2.9%)	p=0.072
	Couple family	2809	101 (3.6%)		114 (4.1%)		46 (1.6%)	
Respondent highest educational level (Sweep 6)	Degree	1084	41 (3.8%)	p=0.503	59 (5.4%)	p=0.201	18 (1.7%)	p=0.896
	Highers	1095	52 (4.7%)		42 (3.8%)		21 (1.9%)	
	Other	1145	46 (4.0%)		52 (4.5%)		22 (1.9%)	
Household income (Sweep 6)	< £15000 pa	494	24 (4.9%)	p=0.524	22 (4.5%)	p=0.938	10 (2.0%)	p=0.953
	£15000-£25999 pa	631	23 (3.6%)		27 (4.3%)		12 (1.9%)	
	£26000-£43999 pa	952	45 (4.7%)		46 (4.8%)		17 (1.8%)	
	≥ £44000 pa	1069	40 (3.7%)		46 (4.3%)		18 (1.7%)	
SIMD Quintile (Sweep 6)	Q1 (Least Deprived)	755	23 (3.0%)	p=0.471	34 (4.5%)	p=0.350	13 (1.7%)	p=0.892
	Q2	763	34 (4.5%)		40 (5.2%)		13 (1.7%)	
	Q3	666	32 (4.8%)		31 (4.7%)		12 (1.8%)	
	Q4	592	26 (4.4%)		31 (5.2%)		14 (2.4%)	
	Q5 (Most Deprived)	553	24 (4.3%)		17 (3.1%)		9 (1.6%)	

Supplemental Table 2: Prevalence of ASD, RAD and overlap, in relation to mother/child characteristics

		N	ASD		RAD/DSED		ASD + RAD/DSED	
			Prev (%)	p-value	Prev (%)	p-value	Prev (%)	p-value
All		3331	139 (4.2%)		154 (4.6%)		61 (1.8%)	
Sex of child	Male	1697	90 (5.3%)	p=0.001	91 (5.4%)	p=0.032	40 (2.4%)	p=0.027
	Female	1632	49 (3.0%)		62 (3.8%)		21 (1.3%)	
Age of mother at birth	< 20	147	14 (9.5%)	p<0.001	15 (10.2%)	p<0.001	5 (3.4%)	p=0.030
	20-29	1169	69 (5.9%)		68 (5.8%)		29 (2.5%)	
	30-39	1861	51 (2.7%)		61 (3.3%)		24 (1.3%)	
	≥ 40	122	2 (1.6%)		6 (4.9%)		1 (0.8%)	
General health (Sweep 6)	Very good	2454	70 (2.9%)	p<0.001	86 (3.5%)	p<0.001	24 (1.0%)	p<0.001
	good	715	46 (6.4%)		49 (6.9%)		25 (3.5%)	
	fair	147	21 (14.3%)		15 (10.2%)		10 (6.8%)	
	bad	13	2 (15.4%)		3 (23.1%)		2 (15.4%)	
Number of accidents reported (Sweeps 1-6)	None	1393	41 (2.9%)	p=0.004	59 (4.2%)	p<0.001	18 (1.3%)	p=0.027
	1-2	1509	74 (4.9%)		62 (4.1%)		30 (2.0%)	
	3-5	384	19 (4.9%)		24 (6.2%)		10 (2.6%)	
	6 or more	45	5 (11.1%)		9 (20.0%)		3 (6.7%)	
Use of smacking reported (Sweeps 2 & 4)	No	1617	61 (3.8%)	p=0.422	65 (4.0%)	p=0.202	30 (1.9%)	p=0.895
	Yes	1597	70 (4.4%)		80 (5.0%)		28 (1.8%)	

Supplemental Table 3 Multivariable predictors of cost. Cost at sweeps 2-4 is modelled using generalised mixed models assuming a log link and Gamma variance. Models identified in forward stepwise variable selection from number of accidents, age of child at each sweep, child sex, age of mother at birth, household level of education at sweep 6, family type, household employment and family type, number of adults in household, number of children in household, income, urban rural classification and SIMD.

	ASD	RAD/DSED	ASD + RAD/DSED	ASD * RAD/DSED
	Estimate (95% CI), p-value	Estimate (95% CI), p-value	Estimate (95% CI), p-value	Estimate (95% CI), p-value
	0.02 (0.01, 0.02), p<0.001		0.01 (0.01, 0.02), p<0.001	0.01 (0.01, 0.02), p<0.001
		0.03 (0.02, 0.04), p<0.001	0.01 (0.00, 0.03), p=0.046	0.02 (0.00, 0.04), p=0.044
				0.00 (0.00, 0.00), p=0.384
1	0.39 (0.34, 0.44), p<0.001	0.39 (0.34, 0.44), p<0.001	0.38 (0.34, 0.43), p<0.001	0.38 (0.34, 0.43), p<0.001
2	0.66 (0.53, 0.79), p<0.001	0.65 (0.52, 0.78), p<0.001	0.65 (0.52, 0.78), p<0.001	0.65 (0.52, 0.78), p<0.001
more than 2	0.84 (0.62, 1.07), p<0.001	0.85 (0.63, 1.07), p<0.001	0.84 (0.62, 1.07), p<0.001	0.84 (0.62, 1.07), p<0.001
	$p^*<0.001$	$p^*<0.001$	$p^*<0.001$	$p^*<0.001$
	0.005 (0.003, 0.006), p<0.001	0.005 (0.003, 0.006), p<0.001	0.005 (0.003, 0.006), p<0.001	0.005 (0.003, 0.006), p<0.001
	-0.08 (-0.13, -0.03), p=0.003	-0.09 (-0.14, -0.03), p=0.001	-0.08 (-0.13, -0.02), p=0.004	-0.08 (-0.13, -0.02), p=0.004
